# New Insight into the Treatment of Advanced Differentiated Thyroid Cancer

**DOI:** 10.1155/2012/437569

**Published:** 2012-12-27

**Authors:** Arash Safavi, Aparna Vijayasekaran, Marlon A. Guerrero

**Affiliations:** ^1^Department of Surgery, University of Arizona, Tucson, AZ 85724-5131, USA; ^2^The University of Arizona Cancer Center, Tucson, AZ 85719-1454, USA

## Abstract

The vast majority of patients with differentiated thyroid cancer (DTC) are treated successfully with surgery and radioactive iodine ablation, yet the treatment of *advanced* cases is frustrating and largely ineffective. Systemic treatment with conventional cytotoxic chemotherapy is basically ineffective in most patients with advanced DTC. However, a better understanding of the genetics and biologic basis of thyroid cancers has generated opportunities for innovative therapeutic modalities, resulting in several clinical trials. We aim to delineate the latest knowledge regarding the biologic characteristics of DTC and to describe the available data related to novel targeted therapies that have demonstrated clinical effectiveness.

## 1. Introduction 

Thyroid cancer, the most common endocrine malignancy, constitutes about 1% of all malignancies. Differentiated thyroid cancer (DTC) includes papillary (PTC), follicular (FTC), and Hürthle cell (HCC) carcinoma, accounting for more than 90% of all thyroid cancers [1, 2].

The incidence of thyroid cancer has been steadily increasing over the last 20 years. In the United States alone, an estimated 56,460 new cases will be diagnosed in 2012; of those cases, 75% will occur in women [3]. In spite of the rising incidence, the death rate has remained relatively constant; in 2012, the American Cancer Society expects a total of about 1,780 deaths [3]. Across the world, the annual incidence of DTC varies, affecting an estimated 1.2 to 2.6 men per 100,000 individuals and an estimated 2.0 to 3.8 women per 100,000 individuals.

Most cases of DTC are slowly progressive; when identified at an early stage, patients can frequently be cured with adequate surgical care and radioactive iodine 131-I (RAI) ablation therapy. But *advanced* DTC—defined as thyroid cancer that has become metastatic, is inoperable, or is refractory to RAI therapy—is associated with a poor prognosis [4].

Cytotoxic chemotherapy and radiotherapy are essentially ineffective for most patients with advanced DTC. Over the last decade, a better understanding of the genetics and biologic basis of thyroid cancers has generated opportunities for innovative therapeutic modalities [5, 6]. In this paper, we aim to delineate the latest knowledge regarding the biologic characteristics of DTC and to describe the available data related to novel targeted therapies that have demonstrated clinical effectiveness.

## 2. Materials and Methods

Using the MEDLINE, Embase, and Cochrane databases, we carried out a literature search. We used the specific terms “metastatic thyroid cancer,” or “advanced thyroid cancer,” and “targeted therapies,” as well as the name of each individual agent. For review and possible inclusion in our study, we selected only relevant articles (describing either clinical experience or trials) published in English from January 2000 through September 2012. In addition, we checked ClinicalTrials.gov for information on registered clinical trials; we searched for any results of trials, using the keywords “thyroid neoplasm” and “randomized,” and then incorporated results we found into our study.

## 3. Results 

### 3.1. Potential Molecular Targets

#### 3.1.1. Papillary Thyroid Cancer (PTC)

Mutations of the B isoform of RAF kinase (BRAF mutations) have been reported in about 40% to 70% of PTC tumors [24, 25]. The *BRAF* ^*T*1799*A*^ mutation is the most common genetic change in PTC, which leads to a V600E amino acid substitution and then to constitutive activation of the BRAF kinase, and hence upregulation of downstream pathways [26].

*RET/PTC* mutations are also well known as one of the most common mutations in PTC. At least 15 types of *RET/PTC* rearrangements have been described [27, 28]. Some studies have underscored that RET rearrangements are associated with the lack of transformation of PTC into poorly differentiated or anaplastic carcinoma [29, 30]. 

*BR* *AF* ^*T*1799*A*^ (V600E) mutations are exclusive to PTC: they are not found in any other forms of DTC or in follicular (FTC), Hürthle cell (HCC), or medullary thyroid cancer [26]. No overlap seems to exist between *RET/PTC, BRAF*, or *RAS* mutations in PTC [31]. On the other hand, *BRAF* ^*T*1799*A*^ (V600E) is a definitive marker of malignancy, as it has never been found in benign nodules while *RET/PTC* has been found in nonmalignant thyroid nodules. The biological and clinical significance of *RET/PTC* in benign thyroid lesions is still controversial [32, 33].

*NTRK1* rearrangements are considerably less frequent in PTC than are *RET* rearrangements. The rate of *NTRK1* rearrangements was about 3% in cases of post-Chernobyl PTC [34]. All these mutations potentially lead to upregulation of the *RAS*/BRAF/mitogen-activated protein kinase (MAPK)/extracellular signal-related kinase (ERK) kinase (MEK)/ERK or MAPK pathway that is involved in thyroid cancer.

#### 3.1.2. Follicular Thyroid Cancer (FTC)

In a comparative study of *RAS* mutations and *PPAR*γ**rearrangements, 49% of conventional FTC tumors had *RAS* mutations, 35% had *PAX8-PPAR*γ** rearrangements, and only 3% had both abnormalities; 12% were negative for both [35]. In some studies, *PAX8-PPAR*γ*1 rearrangement*s have been reported in about 53% of conventional FTC tumors [36–38]. About 20% to 30% of FTC tumors in another study had the *PTEN* mutation [37]. The PI3K pathway may also be activated in a few cases of papillary and follicular cancer [38, 39]. The PI3K-Akt pathway is an alternate pathway to the MAPK pathway. In aggressive DTC and anaplastic thyroid cancer mutations that upregulate, both pathways are common [40].

In a pooled analysis of 229 cases of *RAS* mutations using 39 previous publications, Vasko et al. compared the findings with data from their own cohort of 80 patients. Their analysis showed that mutations of codon 61 of N-RAS (N2) were significantly more frequent in FTC (19%) than in PTC (5%) and significantly more common in malignant (25%) than in benign (14%) tumors. H-RAS mutations in codons 12/13 (H1) were found in 2-3% of all types of tumors, but H-RAS mutations in codon 61 (H2) were observed in only 1.4%, and almost all of them were malignant. K-RAS mutations in exon 1 were found more often in PTC than FTC (2.7% versus 1.6%) [41].

Molecular targeted therapies are being developed to target the aforementioned specific mutations and pathways. Tyrosine kinase inhibitors are especially critical because of their important role in the modulation of growth factor: they signal and interrupt the pathologically activated pathways and thereby affect tumor growth. Each tyrosine kinase compound may or may not be exclusive to a single cascade. However, several interconnections exist between various signaling pathways, so a therapeutic spectrum that is too focused may not be optimal [42, 43].

In addition to targeting specific mutations and pathways, molecular-targeted therapies are being developed to target the blood vessels of the tumor, either by a drug-induced obliteration of already-established vessels or by a prompting of the signaling pathways to commence the growth of new blood vessels. In experimental models, manipulation of vascular functions regulated by vascular endothelial growth factor (VEGF) has blocked the growth of DTC [44]. Numerous VEGF subtypes and VEGF receptors—including VEGFR-1 (FLT1) and VEGFR-2 (KDR)—as well as receptors for fibroblast growth factor (FGF) and platelet-derived growth factor (PDGF) are frequently overexpressed in the vascular endothelium of thyroid cancers and also trigger the MAP kinase signaling pathway [45]. Therefore, therapeutically blocking the function of angiogenesis factors might be an important concurrent approach that will improve the outcome of patients with DTC. 

### 3.2. Therapeutic Compounds

#### 3.2.1. Tyrosine Kinase Inhibitors

Recently completed phase 1 and 2 clinical trials suggest that tyrosine kinase inhibitors confer a clinical benefit in patients with DTC. However, not all of these agents have achieved U.S. Food and Drug Administration (FDA) regulatory approval for the treatment of patients with advanced DTC; few are FDA-approved for the treatment of other carcinomas. The mechanism and outcome of these agents are summarized in Table 1.

### 3.3. Clinical Trials

**Motesanib (125 mg/day)**

The First large, international trial of a tyrosine kinase inhibitor in patients with progressive DTC was a phase 2 study of motesanib. Of 93 enrolled patients, 14% had a partial response; stable disease was achieved in 67% of the patients and was maintained for 24 weeks or longer in 35%. The median progression-free survival (PFS) time was 40 weeks [7]. Interestingly, tumors with BRAF mutations responded better than BRAF-negative tumors, despite the absence of BRAF inhibition by motesanib, suggesting that these BRAF-positive tumors might be more dependent on other signaling pathways. The most common treatment-related adverse events were diarrhea, hypertension, fatigue, and weight loss.

**Axitinib (5 mg twice daily)**

In a multicenter phase 2 study of 45 DTC patients, axitinib induced a partial response in 14 (31%); stable disease was achieved for more than 16 weeks in another 19 (42%) patients. The median progression-free survival for all histologic types of thyroid cancer was 18 months. Reported adverse drug effects were hypertension, proteinuria, fatigue, diarrhea, weight loss, and headache [8].

**Sorafenib (400 mg twice daily)**

Four phase 2 trials have reported on the effect of sorafenib in DTC patients. One trial included 30 DTC patients: 7 had a partial response and another 16 had stable disease [9]. The second trial, a larger one sponsored by the National Cancer Institute, included 52 DTC patients: 12% had a partial response and another 65% had stable disease [10]. The third trial included 31 DTC patients: 8 had a partial response and another 11 had stable disease for more than 24 weeks [11]. The fourth trial included 19 DTC patients: 20% had a partial response [12]. The most common adverse drug effects were hypertension, fatigue, weight loss, palmar-plantar erythema, colon perforation, other skin toxicities, diarrhea, alopecia, and arthralgia [9–12].

With early data showing sorafenib to have promise in DTC patients and with sorafenib being commercially available (in the United States, for patients with advanced renal cell carcinoma or unresectable hepatocellular carcinoma), many clinicians began using it in an off-label manner [12]. In a retrospective analysis of sorafenib in 7 Spanish referral centers, DTC patients who were not suitable for curative surgery, for RAI therapy, or for radiotherapy were treated with sorafenib (400 mg twice daily); 3 of 6 DTC patients had a partial response (median follow-up, 11.5 months) [46]. Marotta et al. assessed the impact of off-label sorafenib in a cohort of 17 patients with progressive DTC refractory to radioactive-iodine.Clinical benefit was obtained in 71% of subjects (30% partial response and 41% stable disease; median followup was 15.5 months). Sorafenib was mostly well tolerated, but a high incidence of fatal events was reported (3 patients died from severe bleeding events and 2 from cardiac arrest) [13]. Now in progress is a phase 3 trial comparing the effect of sorafenib versus placebo on progression-free survival in patients with progressive metastatic DTC not responsive to RAI therapy (NCT00984282).

**Sunitinib (37.5 mg daily)**

In a phase 2 trial by Carr et al. of sunitinib in 28 DTC patients, 1 (3%) had a complete response; 7 (25%), a partial response; and another 14 (50%), stable disease [14]. In a second phase 2 trial by Cohen et al. of sunitinib (50 mg/day, 4 weeks on and 2 weeks off) in 31 DTC patients with progressive metastases, 13% had a partial response; 68%, stable disease [15]. Reported adverse drug effects were fatigue, neutropenia, leukopenia, diarrhea, gastrointestinal (GI) bleeding, supraventricular tachycardia, anemia, dehydration, mucositis, odynophagia, thrombocytopenia, laryngeal edema, hypocalcemia, peripheral neuropathy, and infection [14, 15]. Sunitinib is currently FDA-approved and available in the United States to treat patients with renal cell carcinoma.

**Pazopanib (800 mg daily)**

In a Mayo Clinic phase 2 trial of pazopanib in 37 DTC patients, 49% had a partial response. The likelihood of a response lasting longer than 1 year was calculated to be 66%; the PFS time at 1 year, 47% [16]. Adverse drug effects were elevated liver enzymes, lower GI bleeding, hypertension, weight loss, colonic perforation, diarrhea, oral mucositis, hypoalbuminemia, hypokalemia, abdominal pain, chest pain, headache, cough, and raised creatinine levels. Pazopanib is currently FDA-approved and available in the United States to treat patients with advanced renal cell carcinoma.

**Lenvatinib (E7080) (24 mg daily)**

In a phase 2 trial of lenvatinib in 58 patients, 50% had a partial response (54% for “naïve” or newly treated patients, 41% for pretreated patients); 46%, stable disease. The median PFS time was 13.3 months. The dose was reduced in 39% of patients and withdrawn in 29% [47]. Based on these results, a phase 3 trial will soon be initiated to compare the effect of lenvatinib versus placebo on PFS time in patients with progressive refractory DTC (NCT01321554).

**Vandetanib (300 mg/day)**

Vandetanib is the first and only targeted drug to show evidence of efficacy in a randomized trial. In the first randomized phase 2 study of a kinase inhibitor in DTC patients, 145 patients from 16 centers across Europe were recruited for placebo versus vandetanib arms [48]. The vandetanib patients had a longer median PFS time than the placebo patients (hazard ratio (HR), 0.63; 60% confidence interval (CI), 0.54 to 0.74; one-sided *P* = 0.008). For the vandetanib patients, the median PFS time was 11.1 months (95% CI, 7.7 to 14.0); for the placebo patients, 5.9 months (95% CI, 4.0 to 8.9). The most common adverse events were QTc prolongation, diarrhea, asthenia, and fatigue.

**Sorafenib ((400 mg each morning, 200 mg each evening) and Tipifarnib (100 mg twice daily))**

In a phase 1 trial, 35 DTC patients were treated for 21 days in each 28-day cycle (with 7 days off): of 22 DTC patients, 1 (4.5%) had a partial response and 8 (36%) had stable disease for at least 6 months. The median PFS time for all 35 patients was 18 months [49].

**Cabozantinib (XL184) (140 mg daily)**

In a phase 1 trial, of 15 DTC patients, 8 (53%) had a confirmed partial response, 1 of whom saw marked improvement of a bone-infiltrating lesion; 6 (40%), stable disease. The most common adverse events were diarrhea, hypertension, and palmar-plantar erythrodysesthesia [50].

### 3.4. Other Targeted Therapies

Other targeted therapies that have been tested in trials in DTC patients include cyclooxygenase-2 inhibitors, angiogenesis inhibitors, histone deacetylase inhibitors, proteasome inhibitors, and agents to restore RAI uptake. The mechanism and outcome of these agents are summarized in Table 2.

**Celecoxib (400 mg twice daily)**

Patients were treated for 12 months in this phase 2 trial. Of 32 patients, 23 experienced progressive disease or stopped treatment because of drug toxicity, thus attaining the intent-to-treat study endpoint for celecoxib failure. One patient had a partial response, and another completed 12 months of therapy progression-free. The patient with a partial response was still on celecoxib, along with 7 other patients, when the study was terminated. Adverse drug effects included infection, pain, hypertension, fainting, hypocalcemia, lymphopenia, and anemia [17].

**Vorinostat (200 mg twice daily)**

In a phase 2 study, 16 DTC patients were treated for 2 weeks, followed by 1 week off therapy. No patient achieved a partial or complete response [18].

**Thalidomide (started at 200 mg, increased over 6 weeks to 800 mg or to the maximum tolerated dose)**

28 patients were enrolled who had had various histologic types of thyroid cancer: 18% had a partial response; 32%, stable disease. Outcomes were measured in terms of tumor volume, rather than with RECIST. A partial response was defined as a tumor volume reduction >50%. Most patients required dose modifications because of drug toxicity [19]. The same group of researchers who conducted the thalidomide trial reported results of a phase 2 clinical trial of lenalidomide (25 mg daily), with an interim analysis suggesting similar response rates to thalidomide but with less hematologic toxicity. In that subsequent report, 22% of the patients had a partial response; 44%, stable disease [20].

**Rosiglitazone (4 mg daily for 1 week, then 8 mg daily for 7 weeks)**

In an open-label, phase 2 trial of oral rosiglitazone treatment, 20 DTC patients were enrolled: after treatment, 5 had positive RAI scan results; 5, a partial response (decreased thyroglobulin or positive RAI uptake); 3, stable disease (no change in thyroglobulin and RAI uptake status); and 12, disease progression (increased thyroglobulin levels). By RECIST criteria, no patient, after 3 months, had a complete or partial response to rosiglitazone; on long-term followup, the trial did not result in any clinically significant response [21].

**Bexarotene (300 mg daily)**

In this phase 2 trial, 11 patients received the drug for 6 weeks: 8 had partial restoration of iodine avidity, but there was not much tumor reduction [51]. At 6 months after RAI therapy, 6 patients had progressive disease (defined as an increase >10% in serum thyroglobulin levels and/or an increase >25% in tumor dimensions); 2, stable disease. Bexarotene failed to restore susceptibility to RAI therapy [22].

**Selumetinib (75 mg twice daily)**

In this phase 2 trial, 20 patients received 4 weeks of selumetinib increased iodine uptake in 12 of the 20 patients (4 of 8 *BRAF* MUT; 8 of the 12 other patients). The 8 of those 12 cases achieving sufficient iodine avidity to warrant RAI therapy included all 5 patients known to be *NRAS *MUT and 1 *BRAF* MUT patient. Of the 7 pts who have received RAI, 5 had partial responses and 2 patients achieved stable disease [23].

### 3.5. Patient Recruitment in Clinical Trials

Referral for participation in clinical trials should be pondered for patients with progressive or symptomatic metastatic disease [52]. Various research groups in the United States and Europe are recruiting patients with DTC with radioiodine-negative progressive disease. The list of ongoing trials is summarized in Table 3.

## 4. Conclusion

With the increasing overall incidence of thyroid carcinoma worldwide, the burden of advanced and metastatic disease will rise significantly in the next few decades. Together with new discoveries of the biologic pathways of thyroid cancer, therapies specifically targeting these molecules are being formulated and presented for clinical trials. Despite the plethora of clinical trials in thyroid cancer, treatment choices for those with advanced DTC have remained extremely limited and compromised with limiting toxicities. The introduction of new treatment modalities has generated great excitement in both researchers and clinicians. However, it has to be emphasized that except the recently published Vandetanib trial [48] none of the currently available publications compared the new drug outcomes with another drug; another established therapy, placebo or no treatment control group. Currently based on phase 2 trial results, tyrosine kinase agents have shown encouraging outcomes compared to other available agents and may confer a role in the treatment of progressing and symptomatic metastatic disease. These agents should only be initiated in patients with progressive disease or significant tumor burden in the presence of RECIST targets [53].

The latest revision of RECIST (version 1.1) has incorporated some modification into the initial guidelines [53] such as reduction of number of target lesions, assessment of pathologic lymph nodes, clarification of disease progression, and inclusion of fluorodeoxyglucose positron emission tomography (FDG PET) scan in detection of new lesions, to further optimize the assessment of tumor burden [54]. However, this criteria, was originally established to measure patient's response to cytotoxic chemotherapy and may not sufficiently describe tumor response in genomically distinct subsets of patients treated with specific targeted therapies. New molecular targeted agents often induce growth inhibition rather than tumor regression and this will result in limited objective response rates according to RECIST. Nonetheless, currently, the existing data on molecular imaging (i.e., perfusion or diffusion characteristics, metabolism, and necrosis) in thyroid cancer is not adequately validated to integrate into response assessment criteria and hence, anatomical assessment of tumor burden remains the unsurpassed surrogate endpoint. Despite the limitations of the current RECIST criteria, it is crucial that researchers report their outcomes in a standardized fashion and not according to solely clinical determinants to allow data comparison across diverse demographics and different institutions. 

Future direction towards “personalized” tumor treatment based on genotyping may demand tumor assessment tools such as newly proposed Choi criteria that ultimately include functional and molecular imaging rather than anatomical assessment of tumor size [55]. It is also fundamental to standardize recruitment policies in order to register advanced DTC patients in appropriate phase 1 or 2 trials to foster new compounds or combination therapies that eventually will lead to better management or cure in these patients.

## Figures and Tables

**Table 1:** Trials of tyrosine kinase inhibitors in patients with metastatic differentiated thyroid cancer.

Agent	Motesanib [7]	Axitinib [8]	Sorafenib [9]	Sorafenib [10]	Sorafenib [11]	Sorafenib [12]	Sunitinib [13]	Sunitinib [14]	Pazopanib [15]	Lenvatinib [16]
Mechanism	VEGFR, PDGFR, Kit	VEGFR PDGFR, Kit	BRAF, VEGFR	BRAF, VEGFR	BRAF, VEGFR	BRAF, VEGFR	VEGFR PDGFR, Kit, FL3, RET	VEGFR PDGFR, Kit, FL3, RET	VEGFR PDGFR, Kit	VEGFR1-3, FGFR1-4, RET, KIT PDGFR*β*
*N* enrolled	93	60	30	56	31	34	33	43	37	58
Histology (DTC), *n*	93	45	27	52	27	19	28	37	37	58
Median PFS	40 wks	18 mos	79 wks	15 mos	58 wks	12 mos	12.8 mos	NR	11.8 mos	12.6 mos
Outcome (RECIST)										
CR%	0	0	0	0	0	0	3	0	0	0
PR%	14	31	23	12	25	20	25	13	49	50
SD%	67	42	53	65	34	48	50	68	46	46

BRAF: B isoform of Raf kinase; DTC: differentiated thyroid cancer; PDGFR: platelet-derived growth factor receptor; VEGFR: vascular endothelial growth factor receptor; NR: not reported; mos: months; wks: weeks; RECIST: response evaluation criteria in solid tumors; CR: complete response; PR: partial response; SD: stable disease; PFS: progression-free survival time.

Note: vandetanib is not shown in the table.

**Table 2:** Trials of other targeted therapies in patients with metastatic differentiated thyroid cancer.

Agent	Celecoxib [17]	Vorinostat [18]	Thalidomide [19]	Lenalidomide [20]	Rosiglitazone [21]	Bexarotene [22]	Selumetinib [23]
Mechanism	COX-2 inhibitor	Histone deacetylase inhibitor	Antiangiogenesis	Antiangiogenesis	RAIU restoration	RAIU restoration	RAIU restoration
*N* enrolled	32	19	36	25	25	11	12
Histology (DTC), *n*	32	16	28	25	25	11	12
Outcome	RECIST	RECIST	Tumor volume	Tumor volume	Tumor volume	Tumor volume	Tumor volume
CR%	0	0	0	0	0	0	0
PR%	3	0	18	22	20	0	42
SD%	3	56	32	44	12	18	17

RAIU: radioactive iodine uptake; COX-2: cyclooxygenase-2; DTC: differentiated thyroid cancer; RECIST: response evaluation criteria in solid Tumors; CR: complete response; PR: partial response; SD: stable disease.

**Table 3:** Ongoing comparative drugs trials in thyroid cancer.

Type of thyroid cancer	Intervention	Comparison	Expected end	Current status	Identifier
DTC*	Sorafenib	Placebo	Dec 2013	Ongoing, not recruiting	NCT00984282
DTC	Lenvatinib	Placebo	Jul 2013	Active, not recruiting	NCT01321554
DTC	Cediranib	Cediranib and lenalidomide	Sep 2013	Recruiting	NCT01208051
DTC, MTC**	Everolimus pasireotide	Everolimus or pasireotide	Jun 2014	Recruiting	NCT01270321

*DTC: differentiated thyroid cancer.

**MTC: medullary thyroid cancer.
